# Algorithm of CAD Surface Generation for Complex Pipe Model in Industry 4.0 Background

**DOI:** 10.1155/2022/7062052

**Published:** 2022-04-12

**Authors:** Xiaolei Cheng

**Affiliations:** ^1^Intelligent Information Department, Wanbo Institute of Science and Technology, Hefei 230031, Anhui, China; ^2^Hefei University of Technology, Hefei 230009, Anhui, China

## Abstract

The current pipeline surface generation algorithm cannot get the angle information of the corner of complex pipeline surface, which leads to the poor accuracy of the algorithm, the slow speed of 3D point cloud intelligent mosaic, and the large number of effective points. Therefore, a CAD surface generation algorithm for complex pipeline model under the background of Industry 4.0 is designed, extracting and rendering the wireframe model and extracting background of the complex pipeline video. We obtain the angle information of the corner points of the complex pipeline surface, extract and match the feature of the dense point cloud, and construct the 3D point cloud data mosaic model. The pipe surface is generated by using double-nodal B-spline. The experimental results show that the precision and stability of the proposed method are high. In the early stage, the proposed method uses ISS feature extraction algorithm to extract feature of point cloud data, which improves the positioning accuracy effectively and enhances the 3D point cloud intelligent stitching speed.

## 1. Introduction

Industry 4.0 refers to the Industrial Revolution supported by the Internet and big data, which is the fourth Industrial Revolution derived from the information age. According to the requirements of Industry 4.0, the construction of the factory needs to move forward in the direction of intelligence, establish digital-physical system, make it participate in the purchase of materials, production, and marketing of products and other links, link all links into a whole, comprehensively improve the industrial production efficiency and the degree of product innovation, and then make the Industry get a greater level of progress on the original basis [1, 2]. The strategic goal of Industry 4.0 was put forward by Germany for the first time. At present, China has also realized its importance and has further regulated the pace and direction of industrial development by taking it as a specific standard, so as to indicate a new road for the development of Chinese Industry. With the development of society and economy and the improvement of science and technology, the Internet has become the focus of development and application in various fields.

Tubular engineering structure has the advantages of good mechanical properties and light weight. It is widely used in construction, vehicle engineering, biomedicine, chemical Industry, national defense and military, and other fields, for example, frame structures in buildings, lightweight engineering in the field of vehicles, vascular simulation in medicine, deep-sea drilling, pipeline robots, and other major equipment [3–5]. In the field of computer-aided engineering (CAE), in order to gain a better understanding of the physical properties of objects, it is often necessary to simulate precisely the objects studied. Accurate simulation can shorten the development cycle, reduce costs, and reduce risks, and accurate modeling is the basis of accurate simulation [6, 7]. Most tubular structures are shell structures. Their accurate simulation has important theoretical and practical value.

Reference [8] proposes an automatic DSM generation method based on CAD model. Firstly, the mates feature information of the top-level components in the structural feature tree of CAD model in SolidWorks platform is extracted by SolidWorks API, arranged in a specific order, and stored in the database. Secondly, the geometric relationship between assembled parts is analyzed to determine the influence of various types of fit on the connection relationship between parts, and the corresponding analysis and comparison rules are established. Finally, the design of automatic generation method of pipe surface is successfully completed by using Visual Basic. Reference [9] proposed a surface equivalent segmentation method with user-defined conditions as the core. The user designs a set of conditions in advance according to the segmentation problem and related surface point attributes, calculates the boundary points based on this, searches the boundary points in turn along the growth direction of the boundary edge, and then generates the boundary edge by ordered boundary point spline interpolation. By constructing the boundary edges of the surface and its subpatches, the surface is divided into a group of isosurfaces. In order to ensure the generality of segmentation, an accurate calculation method of initial boundary points and boundary points of sampling units in parameter domain is proposed. Reference [10] proposes a method to obtain point cloud data based on secondary development of UG NX platform. This paper introduces the tools and basic characteristics of UG NX secondary development and comprehensively uses the secondary development tools and Visual Studio 2010 development environment in UG NX10 0 is designed to complete the dialog box of CAD model discretization. This paper expounds the principle and basic flow of CAD model discretization, compiles the callback function in the dialog box, realizes the discretization function, and obtains the point cloud data. Reference [11] designed an oil and gas pipeline data monitoring and analysis model based on big data management architecture. Firstly, they build the overall architecture of the model, collect and sort out the pipeline data through big data support, preprocess it, and determine the number of rows and columns in the associated arrangement. Brillouin sensing technology is introduced. Based on distributed optical fiber sensors and sensing data, optical fiber is used to replace the data composed of hundreds of traditional sensing points to generate feedback sensing sequence. The distance data is generated by calculating the template and optical fiber feedback, and the distance values of the current oil and gas pipeline are determined by relying on the scattering point emission spectrum relationship distance. Through comparison, whether the current pipeline has bending can be determined to realize the monitoring of pipeline data.

However, the above methods can not obtain the included angle information of corner points of complex pipeline surface, resulting in poor accuracy of the algorithm, slow speed of 3D point cloud intelligent splicing, and more effective points of surface splicing. Therefore, the CAD surface generation algorithm of complex pipeline model under the background of Industry 4.0 is designed. The wireframe model of CAD surface of complex pipeline model is extracted and drawn, and the complex pipeline video is collected according to the model to extract the video background. Then the dense point cloud features of pipe surface are extracted and matched, and the 3D point cloud data stitching model is constructed. Finally, the pipe surface is generated by multiple node B-spline. Finally, experiments show that the algorithm has higher accuracy and stability and lower error rate and can meet the needs of CAD surface generation of complex pipeline model.

## 2. CAD Surface Generation Algorithm of the Complex Pipeline Model

### 2.1. Extracting and Drawing the Wireframe Model

**Figure 1:**
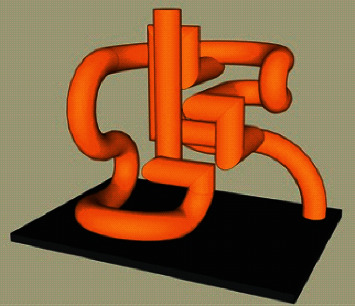
Wireframe model.

Line frame model of geometric modeling system is the earliest technology used to describe object model, which plays an important role in the early development stage of products [12]. It not only can reflect the designer's innovative thinking, but also has the advantages of flexible operation. For example, in the conceptual design of automobiles, in order to shorten the R&D cycle, designers usually draw sketches to represent the shape of the car. In the early stage of underground pipeline design, designers often use wireframe model to replace tubular structure model [13]. The mainstream software has wireframe processing function, which simplifies the tubular model into wireframe model through curve and surface operations such as average, offset, and mixing.  Figure 1 shows the pipe wireframe.

**Figure 2:**
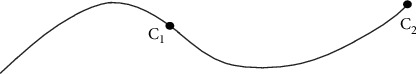
Two-joint pipe.

**Figure 3:**
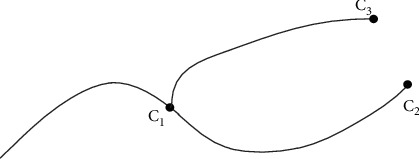
Three-joint pipe.

The extracted wireframe structure is divided into multiple two joints and three joints, as shown in Figures 2 and 3. According to the curve bending degree and actual accuracy requirements, determine the number of segments, as shown in Figure 2, so that the number of curves in each joint is 2, as shown in Figure 3, so that the number of curves in each joint is 3 and the node vector is *U*={0,0,0,0.5, 1,1, 1_*n*_}, and store the geometric information of each joint in the form of text file.

### 2.2. Complex Pipeline Video Acquisition

**Figure 4:**
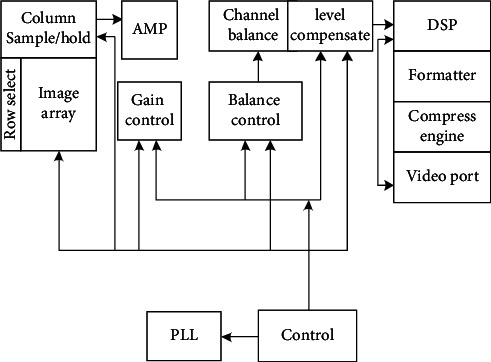
Functional block diagram of the TKF240 acquisition sensor.

TKF240 is selected as the acquisition module of the surveillance camera to complete the functions of the control exposure, image gain, and white balance of the complex pipeline video [14, 15]. The functional block diagram of the TKF240 acquisition sensor is shown in Figure 4.

As can be seen from Figure 4, the photosensitive array of TKF240 acquisition sensor includes 1632 × 1232 pixels, up to 2 million pixels. The video signal processing module of the acquisition sensor is the core of the whole module. The acquisition sensor is used to sharpen the edge of the complex pipeline video image.

In order to ensure the integrity of the video data of complex pipelines, it is necessary to use a video collector for complex pipelines to store the video data of complex pipelines. The 32-bit processor shall be used in the video monitoring system for complex pipelines oriented to Industry 4.0, and the video collector for complex pipelines shall also choose 32-bit processors. Considering that the video collector is larger than 8M to ensure the integrity of the video data of complex pipelines, the SCD985 chip is selected, and the single chip has a capacity of 16 × 16. Since the video data transmission of 32-bit is required in the motion video monitoring system, the 16-× 32 collector composed of two pieces shall be used.

**Figure 5:**
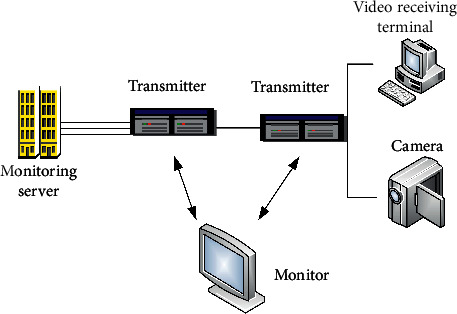
Principle of the Monitor Network Transmission Interface.

If the video image of complex pipeline captured by the monitor camera is displayed in the monitor terminal, it must be realized through the transmission interface of the monitor network. The Ethernet of Industry 4.0 is used to transmit the video image of complex pipeline. The monitoring terminal is used as the server and the video collector of complex pipeline as the client. Monitoring network transmission interface is the data interface connecting complex pipeline video controller and media access controller and can support the independent interface of complex pipeline video [16]. In the system register, the complex pipeline video data transmission is realized by configuring the information technology and computer technology of Industry 4.0. The monitoring network transmission interface is illustrated in Figure 5.

The Monitor Network Transmission Interface allows monitoring applications to access registers. The Monitor Network Transmission Interface based on Industry 4.0 supports multiple input modes and supports complex pipe video images with a maximum input resolution of 8192 × 8192, monitoring network transmission interface with video data scaling and previewing video images and other functions, but also supporting the rotation and flipping functions. Complex piping video surveillance systems can capture complex piping video through the transmission interface of the surveillance network, and surveillance cameras with industrial 4.0 designs have super-high pixels of 3 million to meet the requirements of motion video resolution [17, 18].

Based on the advantages of the Fourth Industrial Revolution, the paper integrates several video processing units in the hardware platform of the monitoring system, collects the image data of the complex pipeline video on the collection sensor, and completes the design of the video collector of the complex pipeline and develops the monitoring network transmission interface of the monitoring system by using Ethernet, completes the design of the monitoring network transmission interface by analyzing the performance and circuit interface of the monitoring network, and realizes the hardware design of the moving video monitoring system [19, 20].

### 2.3. Extracting the Complex Pipeline Video Background

In the context of Industry 4.0, to extract the background of complex pipeline video, we must first determine the picture pixels in the complex pipeline video background. In the complex pipeline video collected by the collector, the value of each pixel follows a specific law. For complex pipeline video monitoring system, due to different monitoring scenes, the background of complex pipeline video usually presents single-mode characteristics [21, 22].

Suppose that, for a complex pipeline video frame sequence *Q*={*q*_1_, *q*_2_, ⋯, *q*_*n*_}, the pixel values in the complex pipeline video together constitute a pixel set *W*={*w*_1_, *w*_2_, ⋯, *w*_*n*_}, so that *μ* is the mean value of pixel set *μ* and *δ* is the variance of pixel set *W*; when the complex pipeline video pixels in *W* conform to the normal distribution, there is

(1) $$\begin{array}{c} W=\frac{1}{\sqrt{2\pi }\delta }e^{-{\left(p-\mu \right)}^{2}/2\delta^{2}}. \end{array}$$

When extracting the complex pipeline video background, the surveillance camera is usually static, and the extracted complex pipeline video background value is the mean value of the statistical value. Assuming that the extracted complex pipeline video background is *ℵ*(*x*, *y*) and the complex pipeline video frames collected by the system are *f*_*i*_(*x*, *y*) and (*i*=1,2,3, ⋯, *N*), the extracted complex pipeline video background is

(2) $$\begin{array}{c} ℵ\left(x,y\right)=\frac{\sum_{i=1}^{N}f_{i}\left(x,y\right)}{N}. \end{array}$$

In a complex pipeline video surveillance system, when the surveillance camera oscillates slightly, the captured complex pipeline video pixels will not follow the normal distribution, and the above method will cause the extracted complex pipeline video background confusion [23]. Based on the normal distribution, a coefficient *α* representing the speed of pixel update is introduced to control the proportion of complex pipeline video background. The updated complex pipeline video background extraction formula is as follows:

(3) $$\begin{array}{c} ℵ\left(x,y\right)=\alpha \left[\mu_{i-1}\cdot f_{i}\left(x,y\right)\right]. \end{array}$$

Because it is difficult for the original complex pipeline video background extraction formula to obey the normal distribution, the complex pipeline video background extraction formula is updated by introducing the coefficient of update speed.

### 2.4. Feature Extraction and Matching of Dense Point Cloud on the Pipe Surface

In the process of reconstruction of complex pipeline surface, the included angle information of corner points of complex pipeline surface is firstly obtained by combining SUSAN corner detection theory, and corner features under contrast of dense point clouds of different pipeline surfaces are extracted [24, 25]. Surface initial matching was carried out on extracted corner point features to obtain the initial matching point set, and the matching results were optimized with the least square median theory. The specific process is described as follows:

In the complex pipe surface template area, the maximum and nonzero point is selected as the corner point, where *g* represents a fixed threshold value in the dense point cloud of the surface, and *ℑ* represents the minimum contrast of corner points that can detect the surface point cloud.

The gray value of complex pipeline surface was analyzed, and the adaptive threshold under contrast *ℑ* of point cloud of different surfaces was extracted, which was expressed by

(4) $$\begin{array}{c} ℑ=a\cdot \frac{\sum_{i=1}^{n}I_{i\max}-\sum_{i=1}^{n}I_{i\min}}{n}, \end{array}$$

where *I*_*i*max_ and *I*_*i*min_, respectively, represent the maximum *i* gray values and the minimum *i* gray values in the point cloud of pipe surface.

Firstly, the initial matching point (*m*_1*i*_， *m*_2*j*_) of the point cloud of the pipe surface is obtained by the correlation method. The two corners are centered on *m*_1*i*_ and *m*_2*j*_, respectively, and *R* is the neighborhood *N*(*m*_1*i*_) and *N*(*m*_2*j*_) of the radius of the pipe surface. Pipe surface point cloud matching points *m*_1_ and *m*_2_ are associated points, and pipe surface is 3 × 3. If *F* in the matrix is the module matrix of two pipe surface point clouds, and the surface point cloud module matrix vector ${\tilde{m}}_{i}$ is the homogeneous coordinate of the matching point *m*_*i*_, there are

(5) $$\begin{array}{c} {\tilde{m}}_{i}^{T}F{\tilde{m}}_{1}=0. \end{array}$$

Assuming that the proportion of wrong matching in the whole pipeline surface point cloud matching set is *ε*, the probability that each pipeline surface point cloud sample contains *U* pairs of correct matching points in multiple pipeline surface point cloud samples is given by

(6) $$\begin{array}{c} U=1-\left.{\left(1-\varepsilon \right)}^{p\cdot m}\right). \end{array}$$

### 2.5. 3D Point Cloud Data Mosaic Model

Set the quadratic pipe surface equation composed of the *i* feature point (*x*, *y*), and the general form is

(7) $$\begin{array}{c} z_{i}=\left\{\begin{array}{c} f\left(x,y\right),\\ a_{0}+a_{1}x+a_{2}y+a_{3}x^{2}+a_{4}y^{2}, \end{array}\right. \end{array}$$

where *a*_0_, *a*_1_, *a*_2_, *a*_3_, *a*_4_ represent the rotation angle. The minimum value is obtained according to the least square principle, namely,

(8) $$\begin{array}{c} \varepsilon^{2}=\sum_{i}{\left(a_{0}+a_{1}x+a_{2}y+a_{3}x^{2}+a_{4}y^{2}-z_{i}\right)}^{2}. \end{array}$$

Derive formula (8) so that its final value is 0. Then, it can be combined with formula (8) to obtain the fitting equation of quadratic pipe surface. The specific expression is as follows:

(9) $$\begin{array}{c} \chi \left(x,y\right)=\left(x,y,a_{0}+a_{1}x+a_{2}y+a_{3}x^{2}+a_{4}y^{2}\right). \end{array}$$

Point cloud mosaic mainly refers to the use of arbitrary transformation to align the point cloud data in two groups of different regions, so that point clouds obtained from points in each region can be accurately matched and spliced [26–28]. Several common point cloud stitching strategies are given as follows:

1. Sequence splicing
2. Global stitching

Three-dimensional topography measurement is mainly composed of control computers, industrial robots, and topography [29, 30]. Among them, the point cloud mosaic model based on the world coordinate system is mainly composed of the following coordinate systems:

1. The coordinate system of the topography sensor is *O*_*S*_ − *X*_*S*_*Y*_*S*_*Z*_*S*_
2. Frame coordinate system composed of rigid connection of topography sensor is *O*_*f*_ − *X*_*f*_*Y*_*f*_*Z*_*f*_
3. The world coordinate system is *O*_*w*_ − *X*_*w*_*Y*_*w*_*Z*_*w*_

Any two coordinate systems between the above three coordinates can be converted, *P* stands for any point within the set range, and the coordinate of this point in the topography sensor coordinate is *P*_*s*_. Through transformation relation _*s*_^*f*^*A*, point cloud measurement data can be obtained and converted to the world coordinate system, and the corresponding relation can be obtained as follows:

(10) Pw=Asw·Asf·Ps.

From the analysis formula, it can be seen that the point cloud data obtained by the robot driving the profilometric sensor at different measuring stations is converted to the world coordinate system, which is constructed on the laser transmitter [31]. Because the transmitter needs to keep the position unchanged in the course of measurement, it is necessary to make sure that the robot can work out the trajectory in advance and measure different positions according to the trajectory.

**Figure 6:**
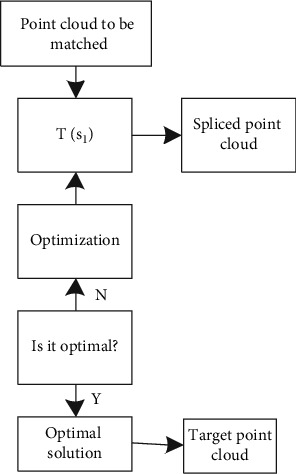
Flowchart of point cloud data splicing.

The swarm intelligence algorithm was used to solve the 3D point cloud data mosaic model [32]: Feature points were extracted for the initial point cloud *s*_1_ and target point cloud *s*_2_ to obtain the simplified model. The swarm intelligence optimization algorithm was used to optimize the evaluation function and obtain the transformation matrix. The obtained transformation matrix is applied to the initial point cloud *s*_1_ to realize the splicing with point cloud *s*_2_. The specific operation process is shown in Figure 6:

1. Set up point sets corresponding to different point clouds, and determine the number of final feature points according to the size of data scale
2. Structured processing of point sets through the K-D tree method to accelerate the acquisition of the distance between point clouds [33]
3. A K-D tree is set up for all points in the source point set *g* by using the target point set to quickly obtain K neighborhood points and calculate the distances between different point clouds and *K* neighborhood points, respectively
4. The regional point sets composed of each point and *K* neighborhood shall be counted, the point expansion matrix shall be established, and the corresponding feature vectors shall be calculated
5. The eigenvalue is evaluated through the eigenpoint extraction algorithm, and the corresponding eigenpoints are extracted
6. The simplified model after extracting the feature points is regarded as a new initial model, and the swarm intelligence algorithm is used to determine the initial parameters and convergence conditions
7. Initialize the optimized individuals to ensure the corresponding spatial location of each search field
8. When an iteration is completed, the current optimal solution and the global optimal solution shall be compared, the better optimal solution shall be set to all new optimal solutions, and step (7) shall be returned, and the above operation process shall be repeated until a required number of iterations are completed
9. Comparing each variable of the final optimal solution with six parameters of the transformation matrix, the transformation matrix is applied to *G*, and obtaining a new point cloud is the point cloud after the completion of the mosaic

### 2.6. Pipeline Surface Generation Based on Double-Nodal B-Spline

In the process of generating pipeline surface, the free deformation technology is used to place the deformed part of the 3D model of pipeline surface parameters into a specific hexahedron space and assign fixed grid parameter coordinates to all points in the hexahedron and on the hexahedron boundary and promote the model to produce deformation effect by adjusting control points. The parametric optimization generation of pipeline surface is effectively realized [34, 35]. The specific steps are as follows:

In the process of parametric optimization generation of pipe surface, it is assumed that *r*_*i*,*j*,*k*_ represents all marked pipe surface mesh control vertices, and the part of the pipe surface parameter 3D model to be modeled is placed in a specific hexahedron space based on *L*_*i*,2_ and *L*_*i*,*N*+1_; then all marked pipe surface mesh control vertices shall meet the following conditions:

(11) $$\begin{array}{c} r_{i,j,k}=U+V\cdot jL_{i,2}+L_{i,N+1}\cdot W. \end{array}$$

Here, *i*, *N*, and *j* represent the boundary of pipe surface mesh control vertices, and *U*, *V*, and *W* represent the coordinates of pipe surface mesh control vertices [36].

In the process of generating pipe surface, the model is deformed by adjusting the control points, and the new coordinates of point *Q* after deformation are calculated by using the following formula [37]:

(12) $$\begin{array}{c} \gamma \left(u,v,w\right)=\frac{b_{i,j}\left(u\right)\cdot b_{j,m}\left(v\right)b_{k,n}\left(w\right)}{r_{i,j,k}\cdot v}. \end{array}$$

Here, *r*_*i*,*j*,*k*_ represents the position of the new coordinates of the control vertex, and *b*_*i*,*j*_(*u*), *b*_*j*,*m*_(*v*), and *b*_*k*,*n*_(*w*) represent the basis function of the pipe surface, respectively, which completes the generation of the pipe surface.

## 3. Experimental Design and Result Analysis

The application effectiveness of the proposed algorithm is verified by the test object with a complex three-tube structure.

### 3.1. Experimental Setup

**Figure 7:**
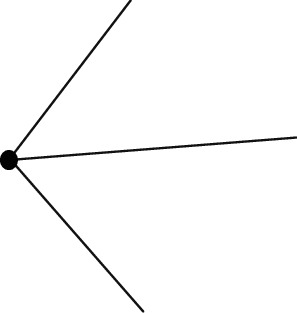
Three straight lines forming 60° to each other.

In this experiment, the three mutual components shown in Figure 7 are used. The tubular surface structure is reconstructed from the wireframe model of the corner. Firstly, the geometric information such as control points and weight factors of the three lines is extracted and stored in the form of files. On the cross section perpendicular to the curve, the weight factor of the control point is *W*={1, 2^1/2^, 1, 2^1/2^, 1, 2^1/2^, 1} and the node vector is *V*={0,0,0,1,1,1}. Figure 7 shows the reconstructed three continuous NURBS surfaces and the corresponding control network, with a radius of 0.3 mm.

**Figure 8:**
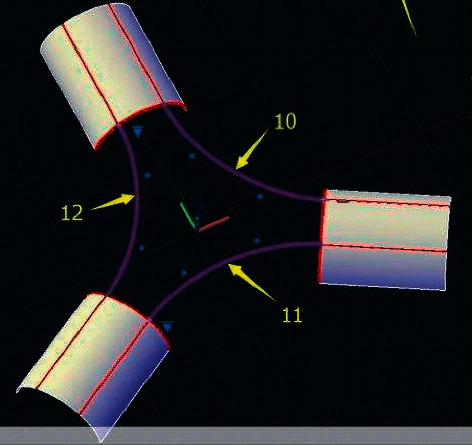
Interface pipe CAD surface generation effect.

According to the mutual formation mode of Figure 7, the CAD surface of the interface pipe shown in Figure 8 is generated by computer software. Among them, Table 1 shows the computer configuration and running time during the experiment.

**Figure 9:**
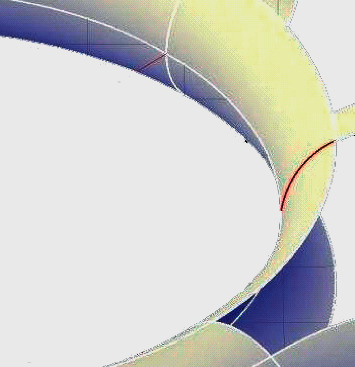
Surface of local pipeline to be reconstructed.

In order to prove the comprehensive effectiveness of the complex pipeline surface reconstruction method based on genetic algorithm, a simulation experiment is needed. In the process of complex pipeline surface reconstruction, firstly, the genetic parameters are given, the initial population size Num = 50, and the maximum number of iterations MAXGEN = 60. Binary coding is used for coding, and the coding accuracy is 0.01. The local pipeline surface to be reconstructed in the first group of reverse engineering is shown in Figure 9.

### 3.2. Analysis of Matching Effect of Surface Reconstruction

**Figure 10:**
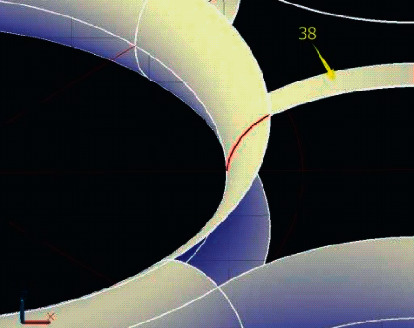
Surface of pipeline surface matching effect in 40 iterations.

The genetic algorithm is used for optimization to find the optimal position for the reconstruction and matching of complex pipeline surfaces. After the coordinate rotation operation, the coordinates of the obtained complex pipeline surfaces are transformed to realize the matching of local complex pipeline surfaces in reverse engineering. The reconstruction and matching effect pictures of local pipeline surfaces when the genetic algorithm is iterated 40 times are given, respectively, as shown in Figure 10.

**Figure 11:**
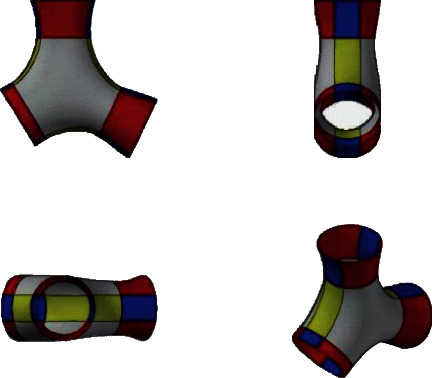
Global complex pipeline surface matching effect.

By analyzing Figure 10, when using genetic algorithm for rough matching of point cloud of complex pipeline surface, with the continuous increase of iteration times, the matching result will eventually converge to near the global optimal solution, which provides a good initial solution for further realizing complex pipeline surface reconstruction. The effect diagram of global complex pipeline surface matching in reverse engineering is shown in Figure 11.

### 3.3. Performance Test

Comparative experiments are carried out by using the automatic surface generation method based on CAD model proposed in [8], the automatic surface generation method based on surface equivalent segmentation method, and the improved algorithm proposed in [9]. Under different experimental times, the accuracy (%), error rate (%), and stability (%) of the surface generation algorithms established by the three methods are compared, respectively. The comparison results are used to measure the overall effectiveness of the three algorithms in establishing the parametric three-dimensional model of pipe surface.

**Figure 12:**
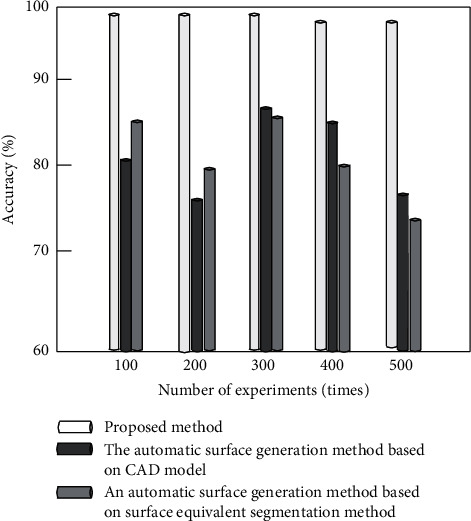
Comparison of accuracy of different methods.

**Figure 13:**
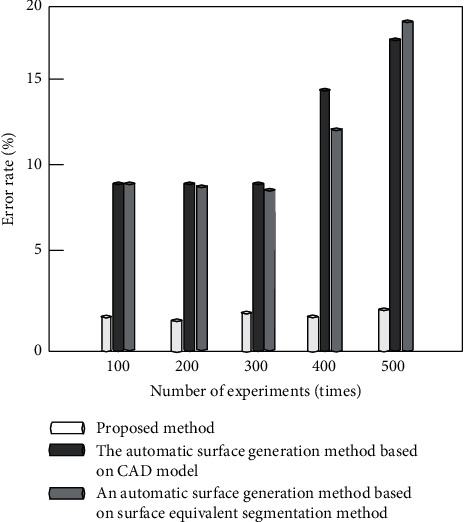
Comparison of error rates of different methods.

**Figure 14:**
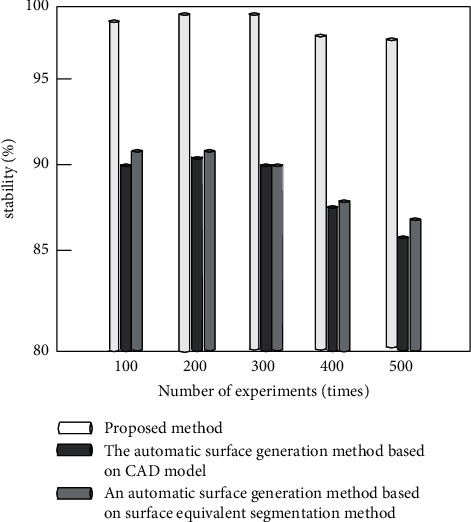
Stability comparison of different methods.

It can be seen from Figures 12 and 13 that the accuracy of the algorithm in this paper is close to 100%, and the error is less than 3%, which is higher than the comparison method. The main reason is that the algorithm in this paper extracts and draws the wireframe model of pipeline surface, extracts the background of complex pipeline video, optimizes the accuracy of surface generation, and reduces the error of surface generation. As can be seen from Figure 14, the stability of the algorithm in this paper is higher than 97%, and the stability of the comparison algorithm is higher than 85%, but in comparison, the stability of the algorithm in this paper is higher. According to the results of Figure 12 and Figure 14, the overall effectiveness of the pipe surface established by the improved algorithm is better than the traditional algorithm. It is also proved that the improved algorithm can splice smooth and continuous pipe surfaces by using curve and surface theory.

### 3.4. 3D Point Cloud Intelligent Splicing Speed Test

**Figure 15:**
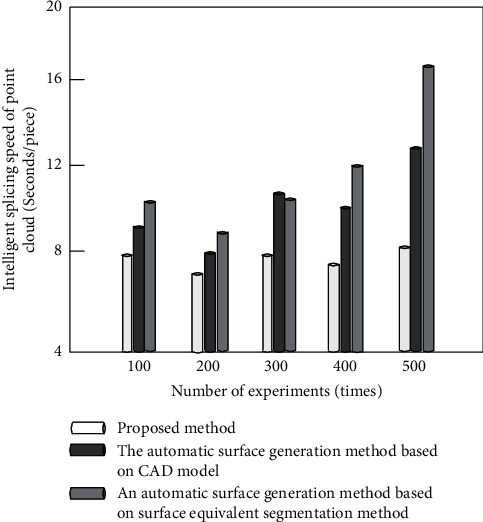
Comparison results of 3D point cloud intelligent splicing speed of different methods.

In order to further verify the advantages of the proposed method, the following experimental tests compare the three-dimensional point cloud intelligent splicing speed of four different methods. The specific experimental comparison results are shown in Figure 15.

By analyzing the experimental data in Figure 15, it can be seen that the proposed method not only effectively improves the positioning accuracy, but also comprehensively enhances the three-dimensional point cloud intelligent splicing speed of the whole method through the ISS feature extraction algorithm in the early stage. Therefore, the splicing speed of the algorithm in this paper is less than 8, and the maximum splicing speed of the comparison method is 16 and 13. Therefore, the 3D point cloud intelligent splicing speed of this method is obviously better than the other three methods.

### 3.5. Test of Effective Splicing Points

The number of effective stitching points is also an important index to verify the comprehensive performance of each method. The higher the number of effective stitching points is, the more ideal stitching results will be obtained.  Table 2 shows the comparison results of the effective points of three different methods.

By analyzing the experimental data in Table 2, it can be seen that, with the continuous increase of rotation angle, the number of stitching effective points of each method is decreasing, but the number of stitching effective points of the proposed method is significantly higher, with a minimum of 137, which is higher than 98 in [8] and 83 in [9], mainly because the proposed method accurately extracts the feature points of point cloud data, effectively promoting the improvement of comprehensive performance of the method.

## 4. Conclusion

In order to optimize the surface reconstruction effect of complex pipeline, the CAD surface generation algorithm of complex pipeline model under the background of Industry 4.0 is designed. On the basis of extracting and matching the characteristics of dense point cloud of pipeline surface under different contrast of dense point cloud of pipeline surface, a three-dimensional point cloud data stitching model is constructed, and the pipeline surface generation algorithm is designed based on multiple node B-spline. The experimental results show that the proposed method has high accuracy and stability, effectively improves the positioning accuracy of surface point cloud data, and comprehensively enhances the intelligent splicing speed of 3D point cloud of the whole method.

## Figures and Tables

**Table 1:** Computer configuration and running time of experimental environment.

Computer configuration	Number of joints	Number of control points	Running time/s
Computer core Quad main frequency 2.66HZ memory 8GB	15	750	5
	25	1290	10
	35	1550	17
	45	2150	25
	55	2800	28
	65	3500	36

**Table 2:** Comparison results of effective splicing points of different methods.

Rotation angle/°	Number of spliced effective points		
	Proposed method	Reference [8] method	Reference [9] method
15	145	121	113
20	140	116	105
25	137	111	97
30	134	107	92
35	130	102	88
40	137	98	83

## Data Availability

The raw data supporting the conclusions of this article will be made available by the author, without undue reservation.
